# Harlequin syndrome in a patient with probable hemicrania continua and exertional headache – is there a link? a case report

**DOI:** 10.1186/s12883-024-03731-y

**Published:** 2024-07-17

**Authors:** Markus Miedl, Philipp Baumgartner, Leah Raffaela Disse, Konrad Peter Weber, Heiko Pohl, Susanne Wegener

**Affiliations:** 1 Department of Neurology, University Hospital Zurich, Zurich, Switzerland; 2 Department of Ophthalmology, University Hospital Zurich, Zurich, Switzerland

**Keywords:** Harlequin syndrome, Hemicrania continua, Trigeminal autonomic headache, Sympathetic nervous system, Exercise (exertional) headache, Horner’s syndrome, Unilateral flushing, Sjögren`s syndrome

## Abstract

**Background:**

The harlequin syndrome is a rare disorder of the autonomic nervous system characterized by unilateral diminished flushing and sweating of the face following exposure to heat or physical activity. It results from sympathetic dysfunction and most commonly occurs idiopathically. A secondary development due to an underlying pathology (e.g., carotid artery dissection, tumors) must be excluded at first appearance. There is evidence that the cranial autonomic system is involved in the pathophysiology of trigeminal autonomic headaches like hemicrania continua. Therefore, an overlap in the pathophysiology of harlequin syndrome and trigeminal autonomic headache disorders seems plausible. However, the association of a harlequin syndrome with hemicrania continua was never reported.

**Case presentation:**

This work describes the case of a 42‐year‐old female patient presenting to our headache unit. The patient reported persisting unilateral headache of the right side of dragging or squeezing character accompanied by trigeminal autonomic symptoms, including lacrimation, nasal congestion, conjunctival injection and Horner’s syndrome, and was responsive to treatment with 75mg/d indomethacin. Five months after the initial consultation, the patient noted that the upper right quadrant of her face was pale after jogging. A harlequin syndrome was diagnosed. Further, she developed a short-lasting, bilateral headache of pulsatile character during strenuous exercise consistent with exertional headache. Comprehensive diagnostic evaluations, encompassing cranial and cervical MRI scans, laboratory tests, and biopsies, culminated in the diagnosis of Sjögren’s syndrome. This finding suggests that the trigemino-autonomic dysfunction may either be idiopathic or a direct manifestation of Sjögren’s syndrome.

**Conclusions:**

This report documents the case of a rare combination of a headache resembling probable hemicrania continua and the harlequin syndrome (and even exertional headache). It illustrates the underlying anatomy of the autonomic nervous system in a clinical context and emphasizes the hypothesis of a pathophysiological link between abnormal sympathetic activity and trigeminal autonomic headaches.

## Background

The harlequin syndrome is a rare condition due to a dysfunction of the facial sympathetic innervation. It was first described in 1988 [1]. The harlequin syndrome is characterized by unilateral paleness and anhidrosis of the face despite exposure to heat, exercise, or emotional stress, where facial flushing and perspiration usually occur [1]. In some patients, the symptoms extend to the arm and upper quadrant of the trunk [1, 2]. Most commonly, this condition is benign without an underlying disease. Still, secondary causes such as carotid artery dissection [3], aneurysm, tumors of the lung apex, neck, and intracerebral tumors must be excluded. Horner’s syndrome can be concomitant with the harlequin syndrome when there is an ocular sympathetic deficit, but this does not necessarily have to be the case [1].

Cranial autonomic dysfunction is also an integral feature of trigeminal autonomic headaches. While other features of cranial autonomic dysfunction, such as Horner’s syndrome, are well described in patients with trigeminal autonomic dysfunction, only 11 cases with harlequin syndrome and a primary headache disorder have been published. Of these cases two patients had cluster headaches [4, 5], eight patients suffered from migraine [2, 6–9], and one patient had post-traumatic headache (with phenotype of tension-type headache) [1]. Four of 11 patients were reported to have an additional primary exertional headache, which, therefore, was more prevalent in this patient collective than in the general population, where its frequency ranges from 1% [10, 11] to 12.3% [12, 13] with female predominance. Here, we report a patient with a combination of harlequin syndrome, a headache resembling probable hemicrania continua, and exertional headache.

## Case presentation

A 42‐year‐old woman sought advice at the emergency department because of continuous pain from dragging or squeezing character behind the right eye and ipsilateral ptosis that had persisted for two weeks. The patient further reported intermittent ipsilateral lacrimation, nasal obstruction, and conjunctival injection. These symptoms appeared suddenly with a severe headache episode and were initially accompanied by nausea, photophobia, and phonophobia for one day. The headache abated about 24 h after onset and persisted at a moderate level, but in the aftermath, transient phases of attack-like aggravation occurred for some hours. In the medical history, the patient described an episode of right-sided peripheral facial nerve paralysis six years ago and an infectious mononucleosis about ten years ago. There was no history of a primary headache disorder. The family history comprised Alzheimer’s disease of a grandfather, an unclassified tremor of a grandmother, and Hodgkin lymphoma of a sister, but no other neurological diseases or headache disorders. The neurological examination confirmed ptosis on the right side and aroused suspicion of an enlarged lymph node on the right side of the neck. Otherwise, it was unremarkable. Laboratory examination and cranial and cervical magnetic resonance imaging (MRI) revealed no pathological findings, including MR angiography and imaging of the orbital structures. No abnormal lymph node was found. A combination of paracetamol, zolmitriptane and oxygen inhalation therapy relieved the pain. A trigeminal autonomic headache was assumed and prophylaxis with 75mg/d indomethacin was established. Four weeks later, the patient reported a positive response to indomethacin with complete remission of the headache, which was diagnosed as probable hemicrania continua.

**Fig. 1:**
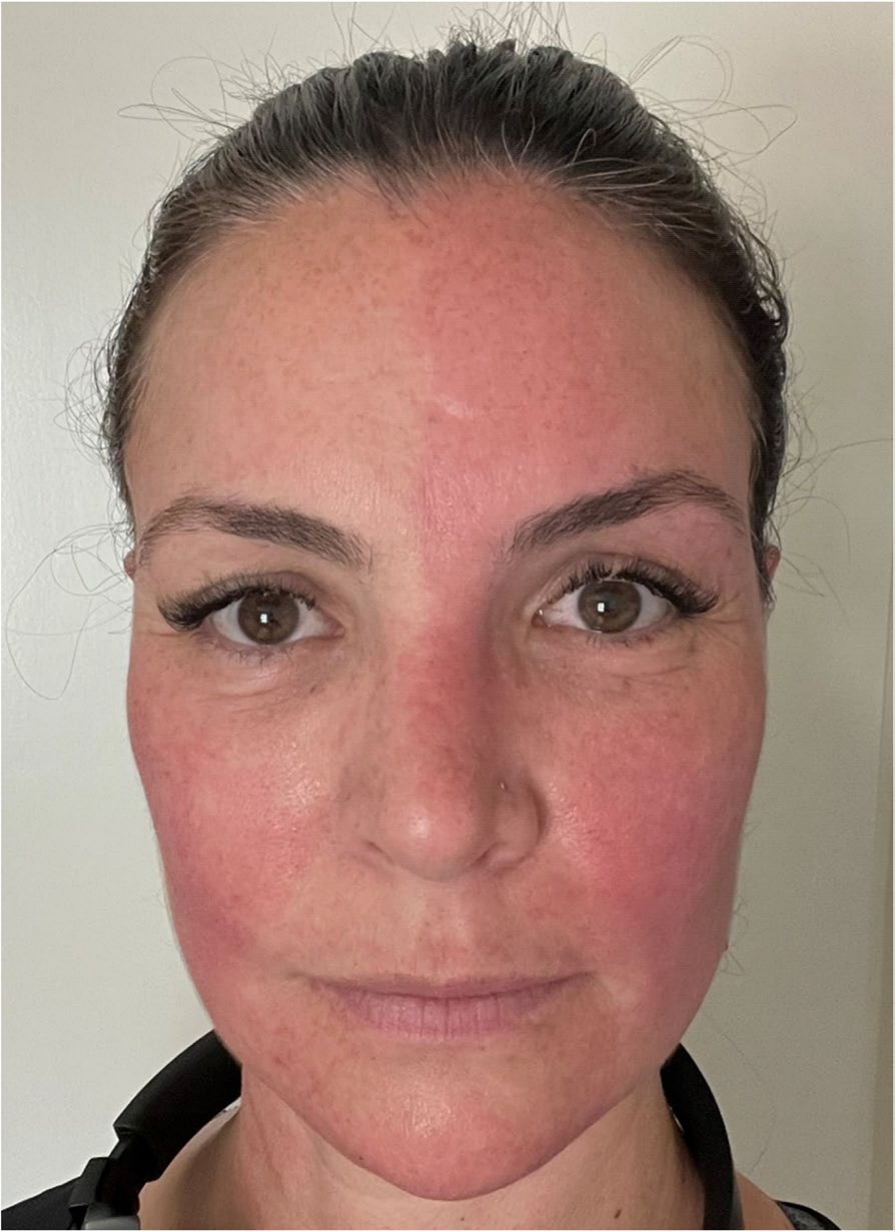
Harlequin syndrome. The patient's face after jogging. You can see the correct presentation with physiological left‐sided flushing of the face and pathological right‐sided absence of flushing, in line with the patient’s statement of right hemicranial headache and paleness of the face. The pathological side is the right side

However, she reported a new type of headache, which only occurred during exercise. She described the pain as having a moderate to high intensity, of a pulsatile character, vanishing within seconds of rest so that no acute pain medication was required. We suspected an exertional headache and increased indomethacin dosage from 75mg/d to 150mg/d. Subsequent diagnostics with 24h-blood pressure measurements ruled out arterial hypertension. Four months after initiation of indomethacin, and overall five months after the onset of her headache, the patient noted the appearance of paleness on the right side of her face, while the left side had turned red after jogging (see Fig. 1).

**Fig. 2:**
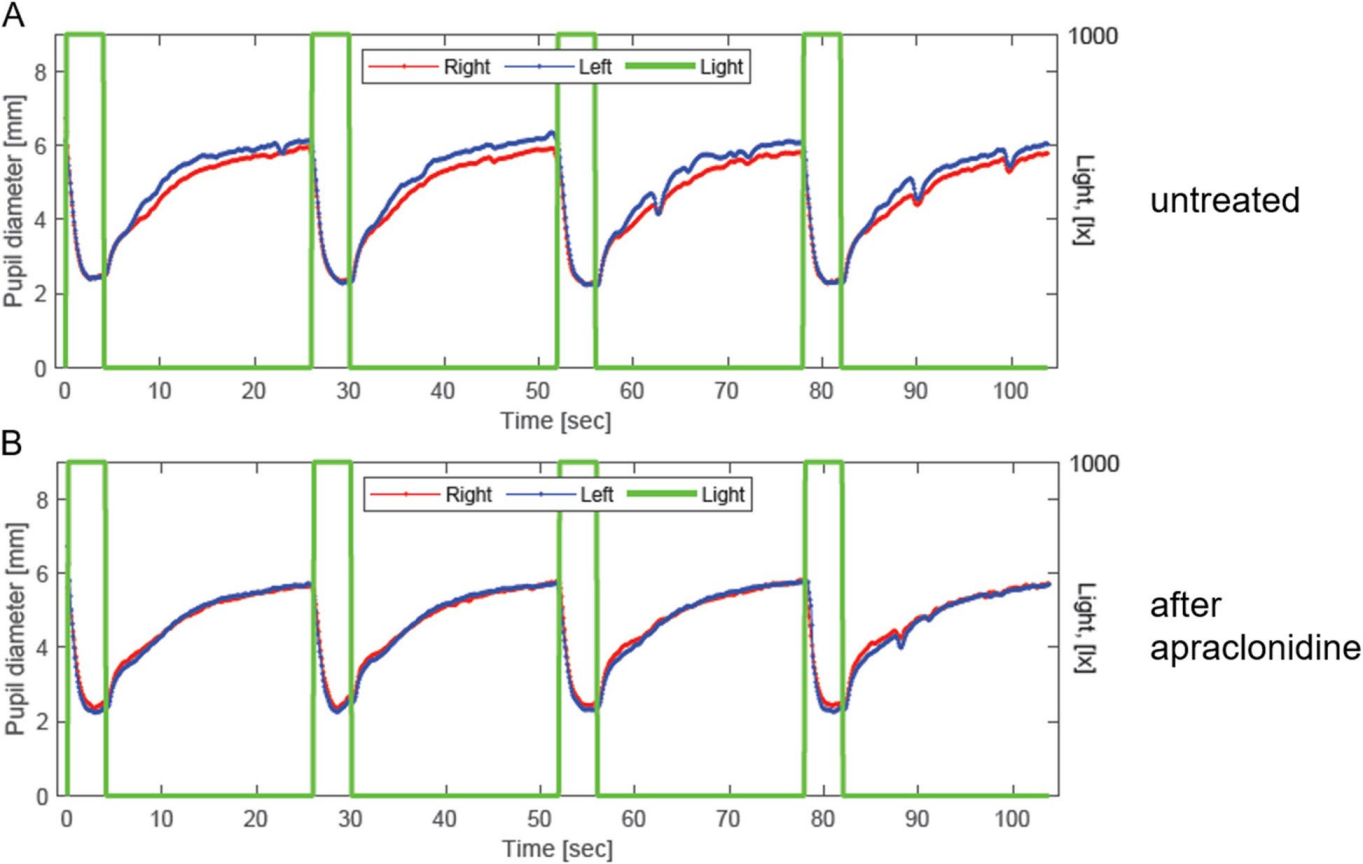
Pupillometry with anisocoria right < left and positive apraclonidine test. In the absence of light, the dilation of the right pupil is less than the dilation of the left pupil due to a loss of sympathetic innervation to the right pupillary dilator muscle, resulting in a subtle anisocoria more pronounced in the dark (A). The pupillary dilator muscle responds to this loss of innervation by increasing its α1 receptors. After topic application of apraclonidine, an α₂-adrenergic receptor agonist and weak α₁-adrenergic agonist, the right, hypoinnervated dilatator muscle reacts hypersensitive with a greater degree of dilation than in the non-denervated left muscle. The equalization of anisocoria is suggestive of Horner’s syndrome in the right eye

While running, she again experienced the short-time exertional headaches described above. On inquiry, she reported the lack of perspiration on the right side of the face during physical activity, so based on the photography and medical history, a harlequin syndrome of the right side was diagnosed. To exclude a secondary cause, MRI, including angiography of the cerebral vessels and carotid arteries, was repeated but again showed no abnormalities, specifically no carotid artery dissection or signs of a pathology along the sympathetic pathway. Additional testing with pupillometry, including apraclonidine testing, was performed to confirm our diagnosis, and a subtle Horner’s syndrome of the right eye was confirmed (see Fig. 2).

In summary, we found a co-incidence of a harlequin syndrome, exertional headache, and a headache resembling probable hemicrania continua, which might indicate a common or at least partial overlap in pathophysiology. Seven months after the onset of the headaches the patient was still in complete remission regarding the hemicrania continua, while exertional headache and the harlequin syndrome were still present. However, ten months after the first onset of symptoms, a right sided headache returned despite indomethacin, which, in the end, was not well tolerated anymore and had to be discontinued. The semiology of the pain changed: It spread from the face towards the neck and was described as a burning quality with the feeling of swelling and stiffness of the neck. Moreover, concomitant to headache, the patient became sensitive to light and noise and intolerant to external stimuli. Besides headaches, she then developed pain diffusely in her body and muscle stiffness, which was worse in the morning, and she described a feeling of numbness and tingling in the feet. The patient was severely burdened by pain until not being able to continue work or follow usual activities of daily life. At that time, she also developed a Sicca syndrome of the eye. Extensive blood tests for autoimmune disorders followed, with positive anti-Ro (SSA) antibody findings and a positive biopsy of minor salivary glands, which led to the diagnosis of Sjögren’s syndrome. To treat neuropathic pain, medication with 900mg/d gabapentin and later 225mg/d pregabalin was prescribed, but this did not relieve the pain. For treatment of suspected peripheral neuropathy related to Sjögren’s syndrome, anti-inflammatory treatment, including prednisone 20mg/d and methotrexate 15mg 1x/week, was started, together with another trial of high-dose gabapentin (1.8 g/d), but this did not alleviate pain symptoms. Thereafter, treatment was adjusted to: methotrexate 20mg 1x/week and duloxetine 60mg/d, while gabapentin and pregabalin were discontinued. The suspicion of concomitant small fiber neuropathy could not be confirmed as skin biopsy and sudometry were negative. The patient was in a reduced overall condition and did not tolerate her medications. After the discontinuation of oral medication (pain medication as well as immune modulating treatments) by the patient herself and adaption of various lifestyle changes, including the reestablishment of sports activities and meditation routine, the patient is now feeling much better. Headache episodes are still present, however, they are now less severe and have features of occipital neuralgia and migraines. The patient was treated once with infiltration of the greater occipital nerve and onabotulinumtoxin A, which had a positive response.

## Discussion and conclusions

Hemicrania continua is one of five subtypes of trigeminal autonomic cephalgias considered by the current International Classification of Headache Disorders 3 (ICHD-3) [14]. Trigeminal autonomic cephalalgias (TAC) disorders share features of unilateral pain and ipsilateral cranial autonomic symptoms, but differ in frequency and duration of attacks and treatment response [15]. Therefore, accurate diagnosis is crucial for effective therapy. The distinct pathophysiology of these primary headache disorders remains to be clearly defined. So far, three parts of the nervous system have been identified to play a role in the development of these primary headache disorders: The trigeminovascular system, the autonomic system, and the hypothalamus [15, 16]. Most data on the pathophysiology of trigeminal autonomic cephalgias are derived from research on cluster headaches. There is a complex interaction between central and peripheral mechanisms. Based on functional imaging studies showing ictal activation of the posterior hypothalamus in all TACs, the hypothalamus is hypothesized to be involved in generating pain, with subsequent trigeminovascular and cranial autonomic activation leading to the typical distribution and accompanying symptoms of these headaches [15]. The positive response to deep brain stimulation targeting the hypothalamus, the chronobiology of TAC like cluster headache, or the decrease of hormones like orexin during attacks supports a key role of the hypothalamus in TAC pathophysiology [17]. The cranial autonomic features of TACs are believed to be due to parasympathetic activation (lacrimation, rhinorrhea, conjunctival injection, and flushing) and sympathetic failure (ptosis, miosis) [18]. The latter is thought to arise from carotid artery wall swelling compressing sympathetic fibers projecting to the orbit [17].

In harlequin syndrome, the underlying pathology of hemifacial loss of flushing is also thought to involve an ipsilateral lesion of sympathetic neurons, leading to disturbed thermoregulatory vasodilatation in the face [19, 20]. Vasomotor and sudomotor neurons originate both from the hypothalamus. As a three-neuron chain, they follow a common pathway via the spinal cord and the sympathetic trunk to the facial target region. Therefore, a lesion in this track not only leads to loss of hemifacial flushing but also includes hemifacial anhidrosis. The postganglionic vasomotor and sudomotor sympathetic fibers (3rd neurons) leave the superior cervical ganglion and travel with the internal carotid artery to innervate the medial forehead and nose, which allows to localize the site of neuronal damage [20]. The forehead and nose were more affected in our patient, which is why a lesion in the course of the internal carotid artery was postulated.

Pain in TACs is mainly mediated through activation of the ophthalmic branch of the trigeminal nerve and release of the signaling molecule calcitonin gene–related peptide (CGRP) [15, 18, 21]. CGRP was shown to cause efficient dilation of the internal carotid artery [22], which could be a possible link to a distension induced sympathetic vasomotor and sudomotor neuron dysfunction. Based on these findings, an overlap in the pathophysiology of primary trigeminal autonomic cephalgias with harlequin syndrome seems plausible. In the present case, the harlequin syndrome occurred after the onset of the headaches and Horner’s syndrome. We hypothesize that the first severe headache episode led to a larger distension of the internal carotid artery with subsequent permanent damage of vasomotor and sudomotor fibres surrounding the artery and that oculosympathetic fibres might be more prone to damage. Alternatively, the first headache resembling a probable hemicrania continua might have been the first manifestation of the Sjogren’s syndrome.

Moreover, this was the time when the patient stated suffering from primary exertional headache (PEH). The pathophysiology of PEH is not well understood, but dysfunction of the arterial and venous system has been discussed [23]. Incompetence of the internal jugular valves could lead to elevated intracranial pressure during Valsalva-like maneuvers, during exercise/sport, and thereby cause headache [24]. Furthermore, an impaired cerebrovascular autoregulation has been proposed, as transient spikes in blood pressure could lead to increases in intracranial pressure and subsequent pain [25]. Due to damage to sympathetic vasomotor neurons, the arterial blood vessels could be abnormally dilated following fluctuations in blood pressure, contributing to the generation of pain. More data is needed about the pathophysiology of primary exertional headache, but as the disease typically has a time-limited course, and many patients don’t seek a doctor’s advice, data are hard to collect [23].

Affection of the nervous system is also a common feature of Sjögren’s syndrome [26], and a trigeminal lesion was described as initial symptom [27]. However, according to neurophysiology and histopathological examination, no peripheral neuropathy could be diagnosed in this patient. Moreover, the lack of response to immunosuppressive treatment argues against a common pathophysiology of Sjögren’s syndrome and the pain in this patient.

In summary, this report documents the rare combination of hemicrania continua and harlequin syndrome together with exertional headache. While a case report does not allow generalizability of our findings, it illustrates the underlying anatomy of the autonomic nervous system in a clinical context. It also emphasizes the hypothesis that there may be a pathophysiological link between abnormal sympathetic activity, trigemino-autonomic headaches, and primary exertional headaches. Future studies should collect evidence of common pathophysiological features through brain imaging and autonomous testing, to advance our understanding of these rare but debilitating conditions.

## List of definitions according to headache classification ICHD-3

### Hemicrania continua (ICHD-3: 3.4)

Unilateral headache fulfilling criteria B-D

A) Unilateral headache fulfilling criteria B-D
B) Present for > 3 months, with exacerbations of moderate or greater intensity.
C) Either or both of the following:
  1. at least one of the following symptoms or signs, ipsilateral to the headache:
    conjunctival injection and/or lacrimation.
    nasal congestion and/or rhinorrhoea.
    eyelid oedema.
    forehead and facial sweating.
    > miosis and/or ptosis.
  2. a sense of restlessness or agitation, or aggravation of the pain by movement
D) Responds absolutely to therapeutic doses of indomethacin.
E) Not better accounted for by another ICHD-3 diagnosis.

### Primary Exercional Headache (ICHD-3:4.2)

A) At least two headache episodes fulfilling criteria B and C.
B) Brought on by and occurring only during or after strenuous physical exercise.
C) Lasting < 48 h.
D) Not better accounted for by another ICHD-3 diagnosis.

## Data Availability

Data can be made available in anonymized form upon reasonable request and separate approval.

## Abbreviations

CGRP: Calcitonin gene–related peptide
ICHD-3: International classification of headache disorders 3
MRI: Magnetic resonance imaging
PEH: Primary exertional headache
TAC: Trigeminal autonomic cephalalgia
CNS: Central nervous system
